# Mutation in enterovirus 71 nonstructural protein 3A increases genome replication fidelity and exhibits attenuated virulence in mice

**DOI:** 10.1128/jvi.01207-25

**Published:** 2025-09-17

**Authors:** Fang Yu, Dongyan Xiong, Yao Zhong, Qiu-Yan Zhang, Cheng-Lin Deng, Zhe-Rui Zhang, Chang Wang, Yang Qiu, Han-Qing Ye, Peng Gong, Bo Zhang

**Affiliations:** 1 State Key Laboratory of Virology and Biosafety, Wuhan Institute of Virology, Center for Biosafety Mega-Science, Chinese Academy of Sciences 74614 , Wuhan, China; 2 University of Chinese Academy of Sciences 74519, Beijing, China; Emory University School of Medicine, Atlanta, Georgia, USA

**Keywords:** replication fidelity, deep sequencing, RNA synthesis, virulence, helix unwinding activity

## Abstract

Increasing numbers of studies have highlighted the important implications of the replication fidelity of RNA viruses for virus replication, pathogenesis, and the development of antiviral drugs and live-attenuated vaccines. However, most of the research has focused on viral polymerase, and little information is available about the potential role of other viral replicase proteins. Here, we demonstrated that the mutations of 3A_V75A_ and 3D_V63A+M393L_ in enterovirus 71 (EV71) conferred high-fidelity phenotypes through deep sequencing of virus populations in cell culture and animal models of infection. The 3A_V75A_, 3D_V63A+M393L_, and 3A_V75A_-3D_V63A+M393L_ high-fidelity variants were highly attenuated in immunocompetent suckling mice. The RdRP enzymology data indicated that the V63A + M393L mutation of 3D^pol^ increased the fidelity of RNA synthesis. The addition of the purified 3AB, either WT or V75A, as previously reported, was able to stimulate the RNA synthesis; interestingly, it effectively enhanced the replication fidelity of WT 3D^pol^. Moreover, the RNA chaperone activity assay showed that V75A 3AB had reduced helix unwinding activity compared with WT 3AB, implying the potential correlation between chaperone activity and fidelity regulation. Together, our results reveal a novel role of the 3A protein in fidelity, providing a basis for the development of antiviral inhibitors and live-attenuated EV71 vaccines.

**IMPORTANCE:**

Numerous viral polymerases in various RNA viruses, such as EV71 3D^pol^, have been reported to be involved in the regulation of replication fidelity, while the role of other viral replicases in this process is poorly understood. In this study, we demonstrate that the 3A_V75A_ variant of EV71 confers increased fidelity and attenuated virulence, and the addition of 3AB, either WT or V75A mutant, can enhance the replication fidelity of WT 3D^pol^ during RNA synthesis. Collectively, this work identifies EV71 nonstructural protein 3A as a previously unrecognized fidelity determinant.

## INTRODUCTION

RNA viruses have high mutation rates, ranging from 10^−4^ to 10^−6^ mutations per round of genome replication due to the lack of the same proofreading system as DNA viruses (1, 2). Such error-prone replication confers RNA viruses the capability of rapidly adapting to changes in environments, thereby influencing virus evolution and pathogenesis (3, 4). However, it still needs to be strictly controlled at an optimal fidelity level, as too high mutation rates may lead to error catastrophe, and too low mutation rates may fail to overcome selection pressure. Manipulating fidelity levels has also been developed as a novel strategy for the rational design of live-attenuated vaccines. Thus, it is necessary to identify important fidelity determinants and investigate the precise mechanism underlying the fidelity regulation.

As a core element for virus replication, RNA-dependent RNA polymerase (RdRP) is the key regulator of replication fidelity, especially for the positive-strand RNA viruses with genomes < 20 kilobases (kb) lacking proofreading and other postreplicative repair mechanisms (5). Multiple RdRP-related fidelity variants have been identified in many viruses, including 3D_G64S_ poliovirus (PV; a fidelity variant with a change of Gly-64 to Ser in the polymerase 3D^pol^) (6), 3D_G64R_/3D_G64T_/3D_L123F_/3D_S264L_ enterovirus 71 (EV71) (7, 8), 3D_M296I_ foot-and-mouth disease virus (FMDV) (9), 3D_F232L_ coxsackievirus B3 (CV) (10), nsP4_C483Y_ chikungunya virus (CHIKV) (11), PB1_V43I_ influenza A virus (12), and nsP12_V553I_ coronaviruses (CoVs) (13). Besides the RdRP, a viral proofreading exonuclease (ExoN) in CoVs, a unique viral protein in large RNA viruses (i.e., > 20 kb), was first identified to be involved in fidelity regulation by excising misincorporated nucleotides or nucleotide analog inhibitors at the 3' end of the nascent RNA (14–17). More recent work demonstrated that the nonstructural protein 2 (nsP2) in CHIKV, a viral helicase/protease, could regulate replication fidelity in concert with the viral polymerase nsP4 (18). Together, the current findings not only emphasize the importance of RdRP for replication fidelity but also underscore the necessity of exploring other viral protein components that regulate fidelity.

EV71 is the causative agent of hand, foot, and mouth disease (HFMD) in infants and children and belongs to the genus *Enterovirus* within the family *Picornaviridae* (19). The genome contains a 7.5 kb positive-sense single-stranded RNA, encoding a large polyprotein that is processed into the following three primary precursors: one structural region P1 (VP4–VP1), and two nonstructural regions P2 and P3 (2A, 2B, 2C, 3A, 3B, 3C, and 3D) (20). The P3 region includes crucial components of the replication complex, in which a functional precursor protein 3CD can be further cleaved to form 3C (viral proteinase) and 3D^pol^ (viral RdRP) (21). 3AB, another precursor protein encoded by the P3 region, is a small, membrane-binding protein containing a core hydrophobic region in 3A that inserts into the membrane, flanked by an N-terminal (the soluble moiety of 3A) and a C-terminal (the 3B peptide) regions. It can be further hydrolyzed into 3A and 3B (also known as VPg, a protein primer for RNA replication) (22, 23). 3A/3AB have multiple functions in virus replication, such as anchoring the viral polymerase 3D^pol^ to the membranous vesicles where replication occurs (24, 25); stabilizing the complex between RNA template, primer, and 3D^pol^ to stimulate RNA polymerase activity (26); and acting as a nucleic acid chaperone to facilitate the proper RNA folding (27, 28). We previously selected two NITD008 (an adenosine analog developed as a drug candidate for dengue virus)-resistant EV71 isolates with mutations mapped to 3A (3A_V75A_) and 3D^pol^ (3D_V63A+M393L_) (29). It was demonstrated that either 3A_V75A_ or 3D_V63A+M393L_ mutation alone could lead to resistance to NITD008, and the combination of 3A and 3D mutations enhanced this resistance further. Given that selection of nucleoside/nucleotide analogs (NAs)-resistant variants is a commonly used approach to discover fidelity determinants, we hypothesize that these mutations may represent new fidelity determinants; in particular, 3A protein may also play an important role in the regulation of viral replication fidelity through an unknown mechanism.

In this study, we quantitatively assessed the changes in fidelity of EV71 NITD008-resistant variants in cell culture systems utilizing deep sequencing across the full-length viral genome. It was demonstrated that the 3A_V75A_ variant manifested a high-fidelity phenotype besides the 3D_V63A+M393L_ variant. The double mutant 3A_V75A_-3D_V63A+M393L_ showed a further increase in fidelity. Consistently, deep sequencing analysis of virus populations in ICR suckling mice revealed that the high-fidelity variants produced less genetically diverse populations within target organs, including the mouse brain and muscle, compared with WT EV71, along with a significant reduction in virulence. To investigate the underlying mechanisms of 3A and 3D^pol^ proteins regulating replication fidelity, we performed an *in vitro* RdRP assay and assessed the effect brought by purified 3AB protein. During RNA synthesis, the V63A + M393L 3D^pol^ exhibited a higher fidelity than WT 3D^pol^, and 3AB protein, irrespective of the mutation, had the ability to increase the replication fidelity of WT 3D^pol^. Moreover, the RNA chaperone activity assay showed that V75A 3AB had a reduced helix unwinding activity relative to WT, which may contribute to the increased fidelity. Our results demonstrate that the 3A protein in EV71 plays an important role in fidelity regulation and adds a new member to the list of fidelity regulators.

## RESULTS

### The EV71 nonstructural proteins 3A and 3D collaboratively regulate replication fidelity in cell culture

**Fig 1:**
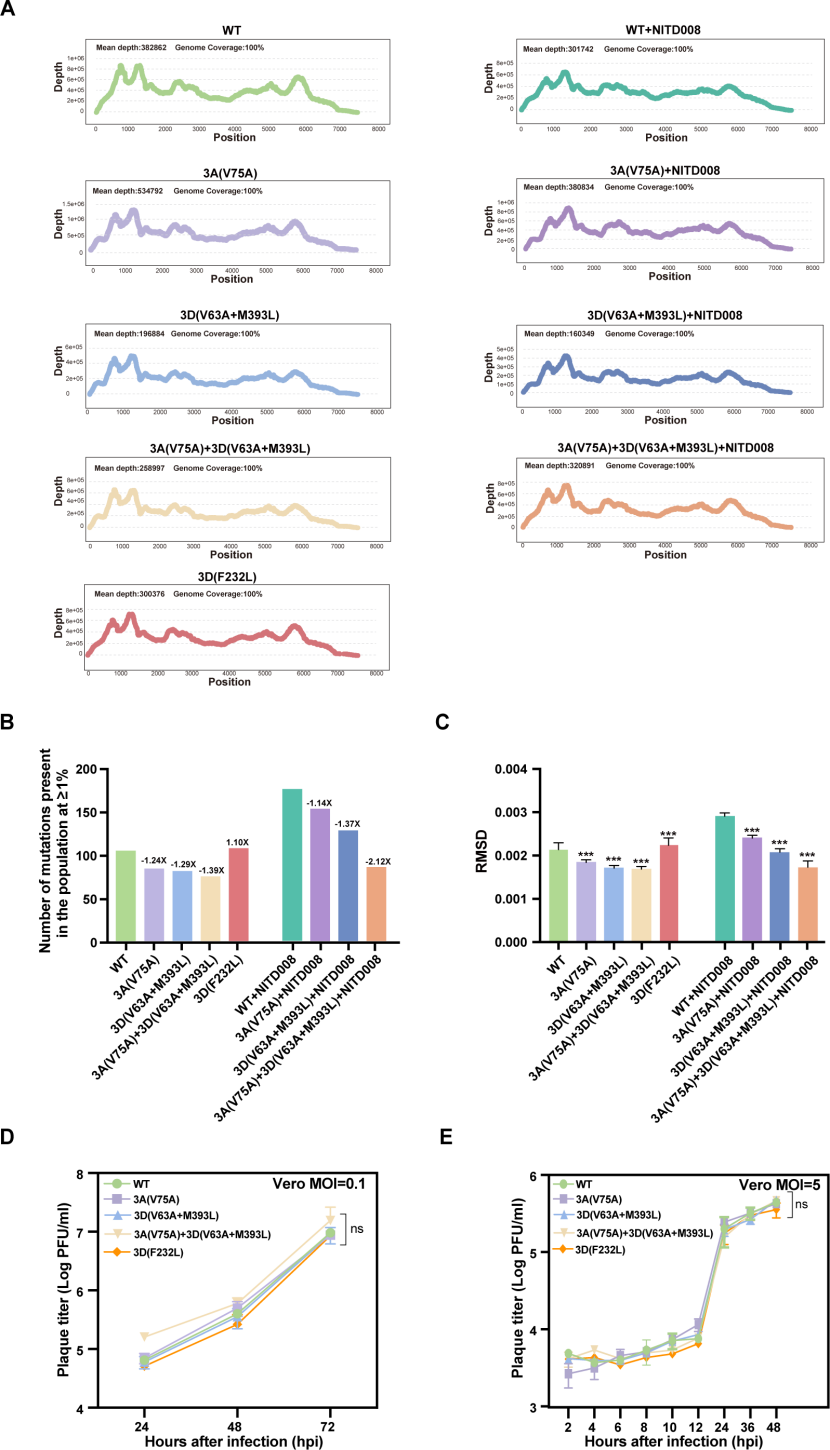
Determination of fidelity from population in the cell culture based on next-generation sequencing. Vero cells were infected with EV71 at a multiplicity of infection (MOI) of 0.01, and viral RNA was collected at 72 hpi. NGS was performed on the DNBSEQ T7 sequencing platform after generating a compatible sequencing library using the Nextera XT DNA Library Preparation kit. The samples were analyzed using the ViVan and PHDtools analysis pipeline. (**A**) Sequencing depth distribution across the whole genome in each variant. The x-axis represents the EV71 genome, and mean depth and genome coverage are indicated at the top of the panel. (**B**) The total number of mutations present in the population with or without 1 µM NITD008 treatment. (**C**) The RMSD distance from each sample to the reference sequence. Mean values ± the standard error of the mean (SEM) are shown (***, *P* < 0.001 [Mann-Whitney U two-tailed test]). (**D**) Comparison of multi-step viral growth kinetics of NITD008-resistant variants and the control virus. Vero cells were infected with viruses at an MOI of 0.1, and viral titers were determined by plaque assay. (**E**) Comparison of one-step viral growth kinetics of NITD008-resistant variants and the control virus. Vero cells were infected with viruses at an MOI of 5, and viral titers were determined by plaque assay. For D and E, significance was determined by a two-way ANOVA test. ns, no significance.

Among the fidelity assessment approaches, deep sequencing of virus population samples is far more informative, as it allows millions or billions of reads to be generated in a single experiment, then the alignment reads showing the amount of minority variants across the whole genome, and the analysis of minority variant diversity can determine fidelity from different populations (13, 14, 18). Thus, to test the role of mutations within 3A and 3D proteins in fidelity, the viral supernatants of P1 WT and variants (3A_V75A_, 3D_V63A+M393L_, 3A_V75A_-3D_V63A+M393L_, as well as a low-fidelity variant control, 3D_F232L_ [10]) harvested at 72 h post-infection (hpi) were subjected to RNA extraction, followed by deep sequencing. Here, the NGS data for minority variants above 1% frequency were mined to calculate the number of minority variants. Under this parameter setting, ViVan generated more conservative mutation profiles that more closely resembled previously reported values for wild-type and low-fidelity variants obtained by molecular clone sequencing (30–32). Meanwhile, all minority variants were aggregated to calculate the RMSD value, which can be used as a metric of viral population diversity (13, 18). In this analysis, the reduction in the number of minority variants or RMSD implies that the virus possesses a high-fidelity phenotype. Each sample achieved a mean depth of more than 100,000 and 100% genome sequence coverage (Fig. 1A).

The inherent fidelity of the variants was first examined. Analysis of the minority variants showed that the 3A_V75A_, 3D_V63A+M393L_, and 3A_V75A_-3D_V63A+M393L_ viruses produced fewer mutations than WT virus, with 1.24-fold, 1.29-fold, and 1.39-fold increases in fidelity, respectively, whereas the control 3D_F232L_ virus resulted in a 1.1-fold decrease in fidelity compared to WT virus (Fig. 1B). Consistently, except for the 3D_F232L_ virus with a higher RMSD value, the RMSD values of 3A_V75A_, 3D_V63A+M393L_, and 3A_V75A_-3D_V63A+M393L_ viruses were significantly lower relative to WT virus, suggesting that these mutations in 3A and/or 3D protein(s) can increase the fidelity of EV71 (Fig. 1C). In parallel, we also compared the fidelity of each variant and WT virus after treatment with NITD008. Both data from the minority variants and RMSD values came to the same conclusion with the fidelity order of WT < 3A_V75A_ < 3D_V63A+M393L_ < 3A_V75A_-3D_V63A+M393L_ (Fig. 1B and C), further confirming the high-fidelity phenotypes of these three variants. In summary, 3A and 3D proteins collaboratively regulate replication fidelity in cell culture.

Besides, we compared the multi-step growth curves and one-step growth curves of WT, 3A_V75A_, 3D_V63A+M393L_, and 3A_V75A_-3D_V63A+M393L_ variants in Vero cells, and no significant differences in viral production were observed between each variant and the WT virus (Fig. 1D and E), further excluding the possibility that increased fidelity in variants resulted from compromised viral replication.

### High-fidelity variants are attenuated in immunocompetent mice

**Fig 2:**
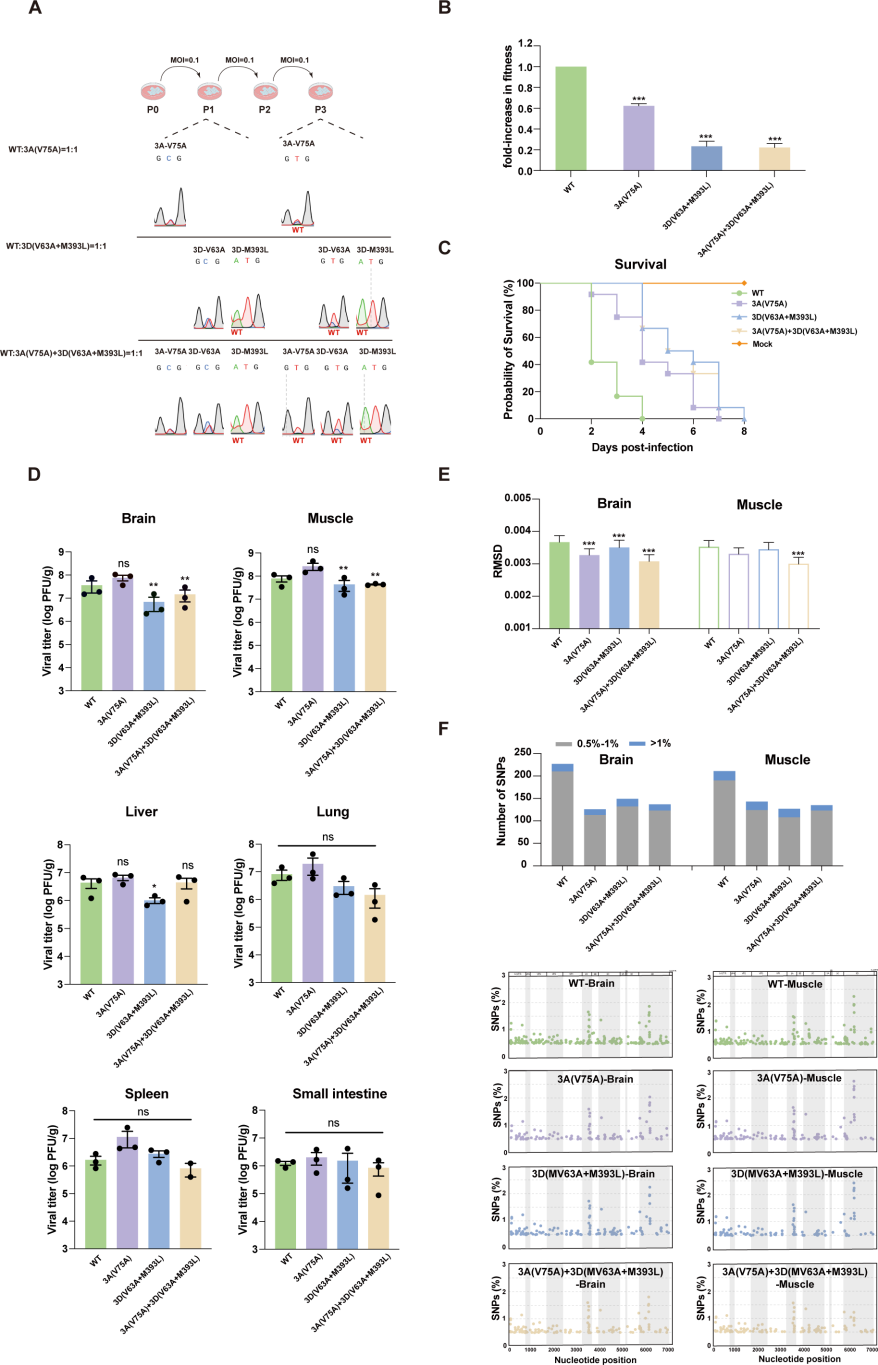
High-fidelity variants are attenuated in 2-day-old immunocompetent ICR suckling mice. (**A**) The abundance of each competitor was measured as the height of the nucleotide encoding either the mutant (3A_V75A_-GCG; 3D_V63A_-GCG; 3D_M393L_-TTG) or the WT (3A_V75A_-GTG; 3D_V63A_-GTG; 3D_M393L_-ATG) in the sequencing chromatograms. (**B**) Competition assays comparing the relative fitness of each variant and the WT virus. Each variant was mixed at a 1:1 ratio with WT virus and inoculated in triplicate into Vero cells at an MOI of 0.1 for three passages. The progeny RNA was RT-PCR amplified to determine the abundance of each competitor. Fitness was measured visually as the height of the nucleotide encoding either the WT (3A_V75A-_GTG; 3D_V63A_-GTG; 3D_M393L_-ATG) or variants (3A_V75A_-GCG; 3D_V63A_-GCG; 3D_M393L_-TTG) in sequencing chromatograms and quantified by ImageJ software. The fitness of the WT virus was defined as 1, and a fitness value of <1 indicated that the high-fidelity variant was less fit than the WT virus. The mean values ± the SEM are shown (*n* = 3) (***, *P* < 0.001 [determined by Student’s t test]). (**C**) The survival curve was monitored. Two-day-old ICR suckling mice were inoculated intraperitoneally with 10^7^ PFU of either WT EV71, or variants (3A_V75A_ EV71, 3D_V63A+M393L_ EV71, 3A_V75A_-3D_V63A+M393L_ EV71), or virus-free cell culture supernatant (mock), *n* = 10 per group. (**D**) The infectious viruses isolated from mice at 4 dpi were measured in PFU per gram of tissue. The mice were sacrificed four days after intraperitoneal inoculation with the 10^7^ PFU virus when the titer reached its peak, *n* = 3 per group. Statistical analyses were performed using a one-way ANOVA test with a Dunnett post-hoc test. *, *P* < 0.05, **, *P* < 0.01; ns, no significance. (**E**) EV71 population diversities in brain and muscle isolates from mice at 4 dpi were measured by RMSD (***, *P* < 0.001 [mean values ± the SEM, Mann-Whitney U two-tailed test]). The mice were sacrificed at four days postinfection, and tissues were subsequently isolated. The RNAs from the tissue were enriched with cDNA amplicons and sequenced after library construction using the Nextera XT DNA Library Preparation kit. ViVan and PHDtools bioinformatics pipeline were used to collect the data per position and calculate the RMSD at each nucleotide position. (**F**) Tissue-specific patterns of diversity in organ-resident populations. The virus was isolated on the 4th day after infection in ICR mice. Diversity is represented as the number and distribution of SNPs.

Due to restricted replication or lower fitness, previous studies have shown that high-fidelity RNA variants are attenuated in animals (3, 6, 33). To assess whether limiting population diversity incurs a fitness cost in cell culture, the adaptability of the WT virus and high-fidelity variants was compared. As expected, the high-fidelity variants did not compete effectively with the WT virus and decreased viral fitness (Fig. 2A and B).

To further evaluate whether these high-fidelity variants were attenuated, we performed infection assays in immunocompetent neonatal ICR mice. Two-day-old ICR mice were inoculated intraperitoneally (IP) in parallel with 10^7^ PFU of WT, 3A_V75A_, 3D_V63A+M393L_, and 3A_V75A_-3D_V63A+M393L_ viruses. Compared with the WT virus, the survival times of three high-fidelity variants were prolonged (Fig. 2C), and the clinical symptoms, such as hind limb paralysis, were weakened (data not shown), presenting attenuated phenotypes. We then determined the viral titers in various tissues, including brain, muscle, liver, lung, spleen, and small intestine, of these variants at 4 days post-infection (4 dpi) (Fig. 2D). There were no significant differences in the viral loads in most of the tissues between these variants and WT viruses, except for a little reduction observed in the brain and muscle of 3D_V63A+M393L_ and 3A_V75A_-3D_V63A+M393L_ variants (one-way ANOVA; *P* < 0.01) and in the liver of the 3D_V63A+M393L_ variant (one-way ANOVA; *P* < 0.05), respectively. To study the intrahost evolution of EV71 high-fidelity variants, viral populations were isolated from the major target tissues (brain and muscle) at 4 dpi and analyzed by deep sequencing to assess the population genetic diversity. In brain and muscle, the diversities of high-fidelity populations were reduced relative to that of WT virus as characterized by RMSD values, although there were no statistical differences in muscle between WT virus and 3A_V75A_/3D_V63A+M393L_ variants (Fig. 2E). Later, single-nucleotide polymorphisms (SNPs) with a frequency of 0.5% or greater detected in mouse were identified to characterize tissue-specific patterns of diversity, which refers to the composition of SNPs found in brain or muscle tissue (Fig. 2F). In the brain, the number of SNPs above 1% from high-fidelity strains is less than that from WT, and this difference is even greater in the number of 0.5%–1% SNPs. In muscle, the high-fidelity variants also had a reduced number of 0.5%–1% and > 1% SNPs compared with WT (Fig. 2F), and there are some non-shared SNPs different from the brain (Table 1; Fig. S1) and Vero cells (Table S1; Fig. S1). In the composition of SNPs across the genome, the diversity patterns of WT populations isolated from brain and muscle tissues were similar, consistent with those of the high-fidelity strains (Fig. 2F). These results indicated that the high-fidelity variants produced less genetically diverse populations in the mouse brain and muscle, consistent with the results of cell culture. Taken together, these results indicated that high-fidelity variants of EV71 were attenuated in the host and exhibited limited tissue-specific patterns of diversity and fitness.

**TABLE 1:** SNPs > 1% in mouse tissues and Vero cells^*a*^

Gene	Amino acid sub	Brain				Muscle				Vero cells			
		WT	3A	3D	3A-3D	WT	3A	3D	3A-3D	WT	3A	3D	3A-3D
5'UTR	C373G	♢				♢							
5'UTR	G385T	♢		♢	♢	♢	□	♢	♢				
5'UTR	A388C	♢		♢		♢	□	♢	□				
VP4	A37P	♢	♢	♢	♢	♢	♢	♢	♢				
2A	G122G (syn)	♢	♢	♢	♢	♢	♢	♢	♢				
2A	L131V (syn)	♢		♢	△	♢	□	♢					
2A	G133G (syn)	♢	♢	♢	♢	♢	♢	♢	♢				
2A	A135G	♢	♢	♢	♢	♢	♢	♢	♢				
2A	V137V (syn)	♢	♢	♢	♢	♢	♢	♢	♢				
2C	Q52H	♢	♢	♢	♢	♢	♢	♢	♢		○	○	○
2C	R100G					□							
3C	G164G (syn)	♢	♢	♢	♢	♢	♢	♢	♢				
3D	A78G	♢	♢	♢	♢	♢	♢	♢	♢				
3D	Y120*	♢	♢	♢	△	♢	♢	♢					
3D	S121C	♢	♢	♢	△	♢	♢	♢					
3D	A122D	♢	♢	♢	♢	♢	♢	♢	♢				
3D	L123*	♢	♢	♢	△	♢	♢	♢					
3D	I125V	♢	♢	♢	♢	♢	♢	♢	♢				
3D	K126N			♢		□	□	♢					
3D	K127*					□	□	□					
3D	K127I							□					
3D	E366G					□	□						

^*a*^ The asterisk represents the stop codon. syn, synonymous mutations. Amino Acid Sub, Amino acid substitution. WT, wild-type strain. 3A, 3A_V75A_ strain. 3D, 3D_V63A+M393L_ strain. 3A-3D, 3A_V75A_-3D_V63A+M393L_ strain. The color-coding scheme is as follows: diamonds, found in both tissues in mice; triangles, found in the brain only in mice; squares, found in the muscle only in mice; circles, found *in vitro* in Vero cells.

### *In vitro* polymerase assay data indicate that the V63A and M393L mutations in 3D^pol^ increase the fidelity of RNA synthesis

**Fig 3:**
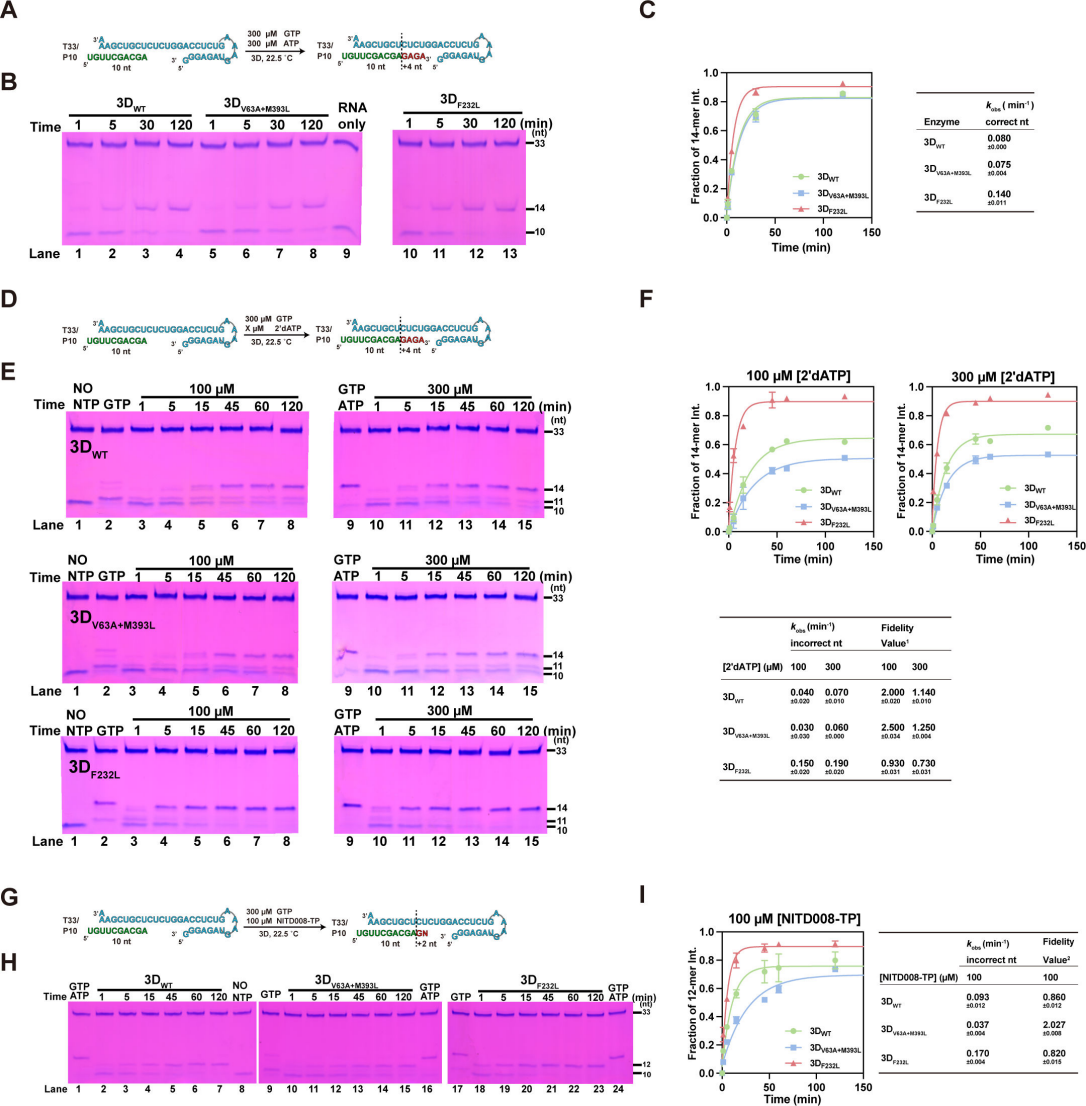
Differences in non-complementary nucleotide incorporation of the EV71 3D^pol^ constructs confirmed the fidelity in the 3D^pol^ mutants. (**A, D, G**) The reaction flow chart and products of polymerase assays. (**B, E, H**) The EV71 3D^pol^ polymerase activities were determined for WT 3D^pol^, V63A + M393L 3D^pol^, and F232L 3D^pol^ when GTP/ATP (**B**), GTP/2′-dATP (**E**), and GTP/NITD008-TP (**H**) were provided as the NTP substrates. (**C, F, I**) The relative intensity of the 14/12-mer product as a function of time was plotted under different NTP substrate(s). *k*_obs_ is the observed rate constant for nucleotide incorporation, estimated by fitting each data set to a single exponential rise model. The fidelity value was calculated as the ratio of the correct *k*_obs_ divided by the incorrect *k*_obs_. *F_1_* = *k*_correct (GAGA)_ / *k*_incorrect (GdA)_ (dA = 2′-dAMP); *F_2_* = *k*_correct (GAGA)_ / *k*_incorrect (GN)_ (*N* = NITD008 MP).

To further investigate how the V63A and M393L mutations affect fidelity, an *in vitro* polymerase assay was performed to evaluate the kinetics of incorporation for cognate and non-cognate nucleotides at relatively saturating nucleotide triphosphate (NTP) substrate concentrations. The T33/P10 RNA construct containing a 33-mer template strand and a 10-mer primer strand was used in a primer-dependent polymerase assay as previously described (34). Briefly, *in vitro* reactions were performed using equal amounts of each purified 3D^pol^. When only GTP and ATP are provided, the primer strand is expected to elongate by four nucleotides (nt) to produce a 14-mer product (Fig. 3A). For both EV71 WT 3D^pol^ and V63A + M393L 3D^pol^, similar RNA elongation profiles of 14-mer products at each time point were shown, and the observed rate constant of correct nucleotide incorporation (*k*_obs_), estimated by the crude accumulation rate of 14-mer under relatively saturating NTP concentrations (300 µM), showed no obvious difference (Fig. 3B and C, 0.080 min^−1^ vs. 0.075 min^−1^). In contrast, the *k*_obs_ of F232L 3D^pol^ was larger than those of WT 3D^pol^ (0.140 min^−1^ vs. 0.080 min^−1^). These data indicate that the V63A + M393L mutation in 3D^pol^ does not have an impact on RNA synthesis when using cognate NTPs as substrates.

To obtain a more quantitative perspective of changes in fidelity, the fidelity values (see legend of Fig. 3) of WT, V63A + M393L, and F232L 3D^pol^ were evaluated. It is known that the substitution of 2′-OH group of NTP ribose with hydrogen (e.g., 2′-dATP and 2′-dCTP) impairs the interaction between NTP and viral RdRP, resulting in a reduction in the efficiency of nucleotide incorporation (35). We first used 2′-dATP to mimic a non-cognate nucleotide and calculated the fidelity value as the correct *k*_obs_ divided by the incorrect *k*_obs_ (Fig. 3D and E). The V63A + M393L 3D^pol^ was on the order of 1.25-fold and 1.1-fold more faithful than WT 3D^pol^ at 100 µΜ and 300 µΜ concentrations of 2′-dATP, respectively (Fig. 3F). Under the same conditions, the fidelity of the control F232L 3D^pol^ was reduced by 2.15-fold and 1.56-fold, respectively, consistent with the previous study (10). Furthermore, the fidelity value was evaluated by monitoring the utilization of the NTP form of NITD008 (NITD008-TP; a chain terminator). By adding GTP and NITD008-TP to initiate the reaction, the incorporation of NITD008 monophosphate (NITD008-MP) can be monitored over time by the accumulation of 12-mer product (Fig. 3G). As observed in Fig. 3H and I, V63A + M393L 3D^pol^ consistently incorporated less NITD008-TP over time, with a 2.36-fold higher fidelity value than WT (Fig. 3I), whereas the fidelity value of F232L 3D^pol^ was 1.05-fold decrease relative to WT 3D^pol^, confirming the increased fidelity of V63A + M393L 3D^pol^.

**Fig 4:**
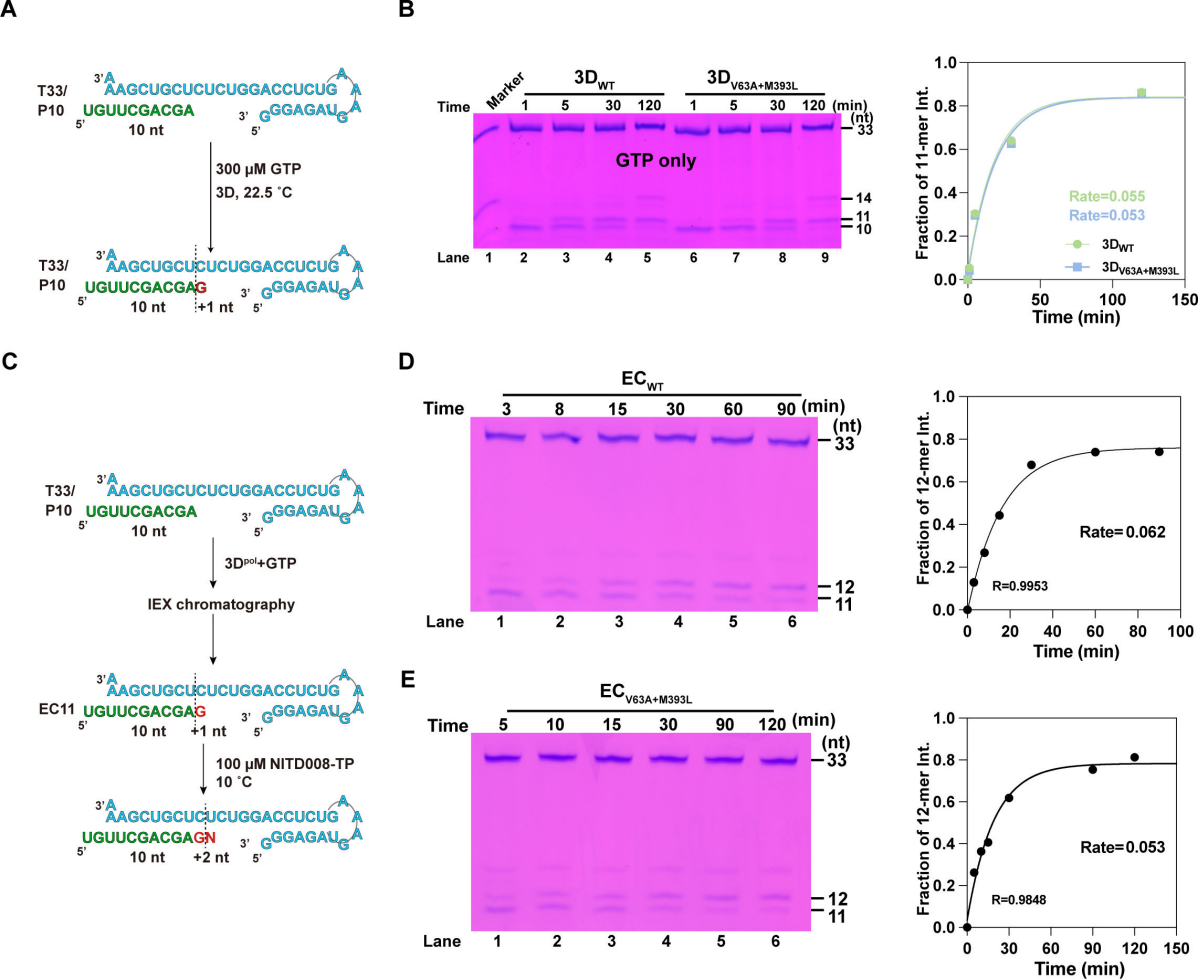
The GTP incorporation assay of 3D^pol^ and the single-nucleotide incorporation assay of EC. (**A, C**) The reaction flow chart and products of polymerase assays. (**B**) The EV71 3D^pol^ polymerase activities were determined for WT 3D^pol^ and V63A + M393L 3D^pol^ when GTP was provided as the NTP substrate. The relative 11-mer product intensity (calculated as the intensity of products of 11 divided by the total intensity of products and primers) as a function of time was plotted for all constructs under GTP substrate. (**D, E**) The accumulation of elongation products was monitored over time for the WT EC and V63A + M393L EC. The relative 12-mer product intensity (calculated as the intensity of products of 12 divided by the total intensity of products and primers) as a function of time is plotted for all constructs under GTP substrate.

Additionally, GTP-only incorporation was tested, and similar accumulation rates of 11-mer products were observed between WT and V63A + M393L 3D^pol^ (0.055 min^−1^ vs. 0.053 min^−1^), indicating the first-step GTP incorporation had little effect on the incorporation rate of the subsequent NTPs or mismatching/non-cognate NTPs, including NITD008-TP in multi-step reactions (Fig. 4A and B). To further accurately determine the differences of NITD008-TP discrimination between WT and V63A + M393L 3D^pol^, a single-nucleotide incorporation assay was performed. As described in Materials and Methods, driven by the polymerase, a "G" is incorporated into the RNA template/primer duplex to form elongation complex (EC, with an 11-mer product), which is then purified and used to determine the incorporation rate of NITD008-TP by monitoring the generation of 12-mer products from U: NITD008-TP mismatch (Fig. 4C) (36, 37). As shown in Fig. 4D and E, V63A + M393L EC yielded a 12-mer product at a slower rate than WT EC. Collectively, these data suggest that the V63A + M393L mutation increased the ability in mismatch discrimination of 3D^pol^ during RNA synthesis.

### 3AB protein regulates the fidelity of RNA synthesis catalyzed by WT 3D^pol^, besides stimulating the activity of polymerase

**Fig 5:**
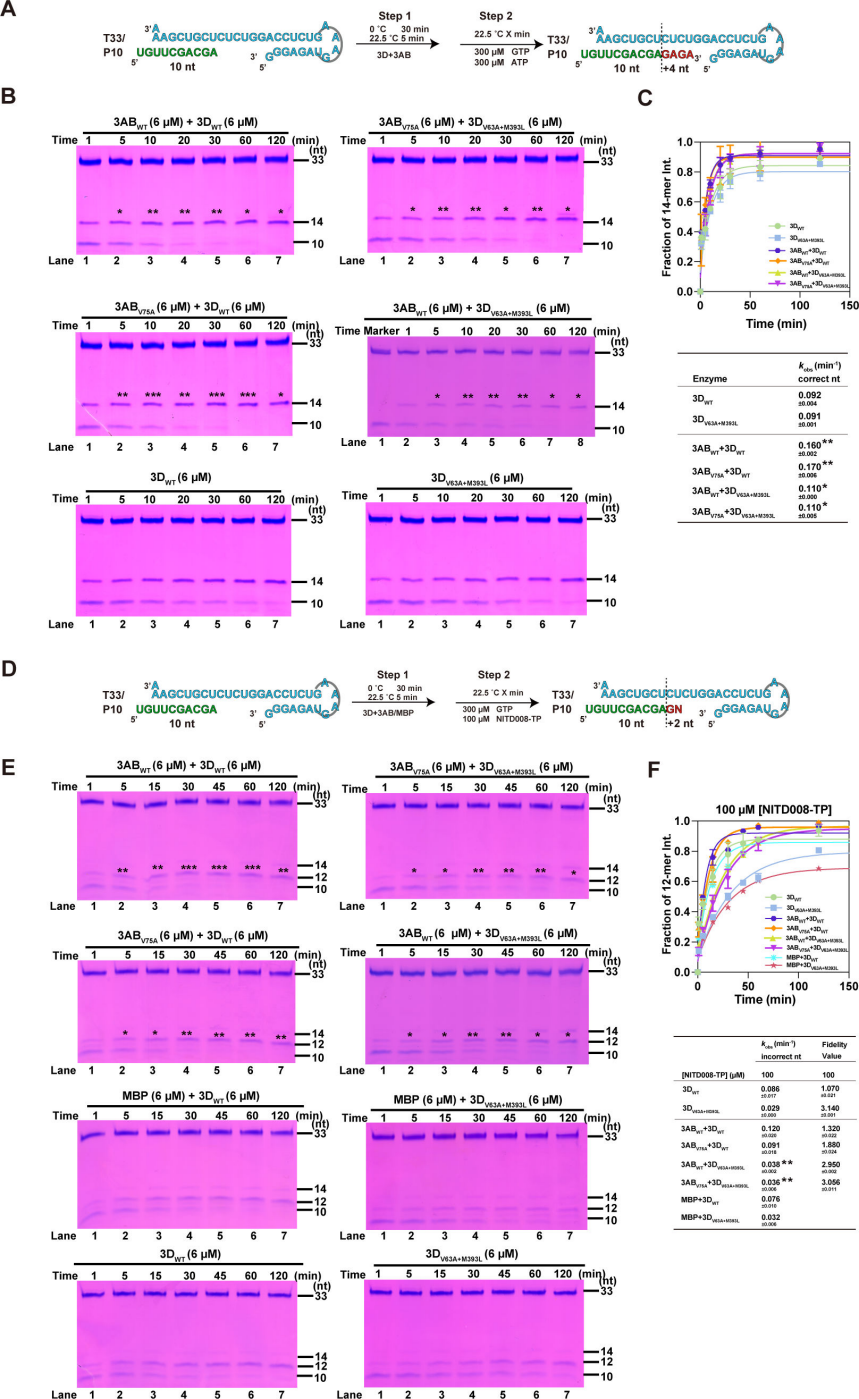
The stimulatory effect and fidelity regulation of 3AB on RNA synthesis by 3D^pol^ in complementary and non-complementary nucleotide incorporation. (**A, D**) The reaction flow chart and products of polymerase assays. (**B, E**) The accumulation of elongation products was monitored over time for the WT 3D^pol^ and V63A + M393L 3D^pol^ constructs. Statistical analyses of the ratio of 14/12-mer product were performed by a two-way ANOVA test followed by the Sidak post-hoc test. *, *P* < 0.05; **, *P* < 0.01; ***, *P* < 0.001; ns, no significance. (**C, F**) The relative 14/12-mer product intensity (calculated as the intensity of products of 14/12 divided by the total intensity of products and primers) as a function of time was plotted for all constructs under GTP/ATP and GTP/NITD008-TP substrate(s). *k*_obs_ is the observed rate constant for nucleotide incorporation, estimated by fitting each data set to a single exponential rise model. The fidelity value was calculated as the ratio of the correct *k*_obs_ divided by the incorrect *k*_obs_. Data are representative of two replicate experiments. The mean values ± the SEM are shown; significant differences of the *k*_obs_ between with and without 3AB in the context of 3D^pol^ were indicated by *P < 0.05 or **P < 0.01 by Student’s *t*-test.

In order to explore the regulation mechanism of 3AB protein on fidelity, we first studied the effects of 3AB on 3D^pol^-catalyzed U: NITD008-TP mismatch using an *in vitro* polymerase assay. It has been demonstrated that the 3A and 3B regions of 3AB have a synergistic effect to stimulate 3D^pol^-catalyzed RNA synthesis *in vitro* (38); hence, the 3AB protein was used in the following assays. To improve the solubility of 3AB protein, the maltose-binding protein (MBP) tag was added at the N-terminus of 3AB (MBP-3AB) to facilitate its folding. Meanwhile, the control MBP-only protein was also expressed and purified. The RNA products of different combinations between WT and mutant in 3AB and 3D^pol^ proteins were measured according to the reaction flow chart (Fig. 5A). As previously reported (26, 39), compared with 3D^pol^ protein alone, the addition of 3AB protein significantly stimulated the yield of 14-mer RNA products to varying degrees in four combinations (Fig. 5B, lanes 2 to 7; *P* < 0.05, two-way ANOVA). In order to further quantify the stimulatory effect of 3AB on the RNA synthesis by 3D^pol^, the *k*_obs_ values of 3D^pol^ with and without 3AB in the correct nucleotide incorporation assay were analyzed. Both WT and V75A 3AB could stimulate the polymerase activity of WT and V63A + M393L 3D^pol^ (*P* < 0.05 for all, Unpaired t test; Fig. 5C), and no significant difference was observed between WT and V75A 3AB, indicating that the V75A mutation did not affect the activity of 3AB to stimulate 3D^pol^-catalyzed RNA synthesis.

To further investigate whether the addition of 3AB protein would regulate the fidelity of 3D^pol^, the ATP substrate was replaced with NITD008-TP in the same reaction (Fig. 5D). As shown in Fig. 5E, the yields of 12-mer RNA product catalyzed by WT/mutant 3D^pol^ were significantly accelerated at different times when adding either WT or V75A 3AB protein (lanes 2 to 7; *P* < 0.05, two-way ANOVA), although there were no significant differences for the *k*_obs_ between with and without WT 3AB in the context of WT 3D^pol^ (Fig. 5F). By comparing the fidelity values for each group, it was shown that the addition of WT and V75A 3AB greatly increased the fidelity of WT 3D^pol^ (1.320/1.070 vs. 1.880/1.070), but had little effect on the fidelity of V63A + M393L 3D^pol^. Besides, the control MBP protein had no effect on the activity of polymerase (Fig. 5F). At the same time, we also followed the same strategy to study the effects of 3AB protein on the incorporation of NITD008-TP in the 11-mer EC reaction system by pre-incubating EC with 3AB (Fig. S2A), but no obvious stimulatory effect was observed (Fig. S2B and C), which confirmed the previous report that 3AB does not stimulate 3D activity on a template that is stably base paired to a primer (39). These results indicate that both WT and V75A 3AB are able to increase the fidelity of RNA synthesis catalyzed by WT 3D^pol^.

### The V75A mutation reduces the nucleic acid helix-destabilizing activity of 3AB protein

**Fig 6:**
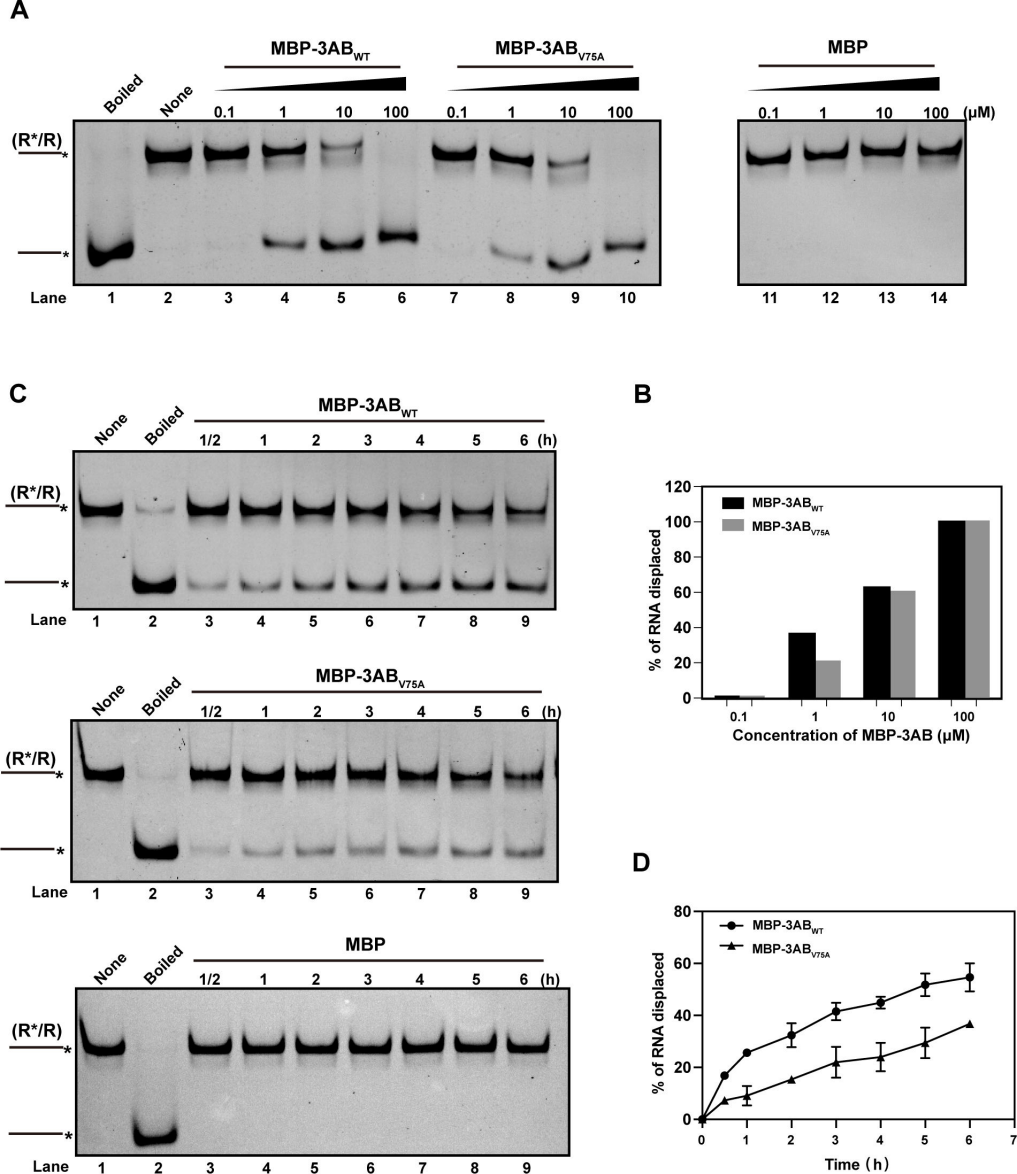
Purified 3AB carrying the V75A mutation has reduced RNA helix-unwinding activity *in vitro*. (**A**) One-hour endpoint helicase assay. Purified proteins of different concentrations were incubated with dsRNA substrate for 1 h. MBP protein was used as a negative control (lanes 11–14). (**B**) Quantification of kinetic analysis in panel A. (**C**) Kinetic analysis of wild-type 3AB and V75A 3AB helicase activity. Purified proteins and dsRNA substrates were incubated at 37°C, and aliquots were removed at the indicated time points. (**D**) Quantification of kinetic analysis in panel C.

It has been reported that 3AB functions as a nucleic acid chaperone with helix-destabilizing activity (27, 28). To address whether the high-fidelity phenotype of the 3A_V75A_ variant is correlated with the chaperone activity of the 3AB protein, an *in vitro* nucleic acid helix-unwinding assay was performed to investigate the effect of the V75A mutation on the RNA chaperone activity of 3AB. Different concentrations of purified 3AB proteins were added to the nucleic acid helix unwinding reaction consisting of hexachloro fluorescein (HEX)-labeled RNA substrates to initiate the reaction, and the yield of unwinding double-stranded (ds) RNA was determined after 1 h by native-PAGE gel electrophoresis followed by Typhoon imager scanning. In comparison with WT 3AB, V75A 3AB exhibited a slower helix unwinding activity, especially at a low concentration (1 µΜ) (Fig. 6A and B). Subsequently, the kinetics of dsRNA unwinding in WT and V75A 3AB-initiated reactions were measured at a concentration of 1 µΜ by calculating the percentage of unwound RNA at each time point (Fig. 6C). Compared with WT 3AB, the V75A 3AB displayed a slower helix unwinding activity (0.450 h^−1^ vs. 0.060 h^−1^, a single exponential equation; Fig. 6D), implying a potential relationship between decreased RNA chaperone activity and high-fidelity regulation of 3AB mutant.

## DISCUSSION

### Strong evidence from cell culture and animal assays indicates that 3A, in concert with 3D^pol^, is an important fidelity regulator

In the presence of nucleotide analogs, the factors affecting replication fidelity can often be identified by passaging the virus population to screen for specific amino acid substitutions in resistant variants. Using the same passaging strategy, we previously selected NITD008-resistant variants of EV71 with 3A and 3D^pol^ mutations (29). It thus prompted us to further investigate whether these mutations are involved in fidelity regulation. As demonstrated by deep sequencing analysis, all these mutations, including 3A_V75A_, 3D_V63A+M393L_ and 3A_V75A_-3D_V63A+M393L_, resulted in high replication fidelity, featured with (i) 1.24-fold, 1.29-fold, and 1.39-fold lower mutation frequency in the 3A_V75A_, 3D_V63A+M393L_, and 3A_V75A_-3D_V63A+M393L_ populations of cell culture (Fig. 1); (ii) a significant reduction in the sequence diversities and tissue-specific patterns of diversity in brain and muscle tissues relative to WT virus (Fig. 2). Consistent with previous reports that alterations of fidelity always result in inefficient adaptation and virulence attenuation (3, 32, 33), the high-fidelity 3A_V75A_, 3D_V63A+M393L_, and 3A_V75A_-3D_V63A+M393L_ variants exhibited reduced fitness and significant attenuation in immunocompetent suckling mice. These data provide strong evidence that the EV71 nonstructural proteins 3A and 3D^pol^ collaboratively regulate replication fidelity, highlighting their potency as a vaccine candidate.

In terms of the potential mechanism underlying the observed attenuation of such mutants, as we know, there are currently the following opinions, most of them based on the studies on the well-characterized high-fidelity PV 3D_G64S_ variant exhibiting decreased virulence: (i) the PV 3D_G64S_ variant reduces viral fitness under a defined selective pressure, making it likely that the reduced spread in mouse tissue could be caused by the increased fidelity of the viral polymerase (3); (ii) it is also considered that the virulence of this variant is determined by the interplay between different variants within the quasispecies that may facilitate expansion and replication in mice (6); and (iii) the replicative speed is more decisive for viral virulence than genetic diversity, as demonstrated by the fact that the compensation for the growth defect of PV 3D_G64S_ restored virulence, whereas compensation of the fidelity phenotype did not (40). In our study, different from the growth defect caused by the G64S mutation in PV 3D^pol^, the mutations in either EV71 3A or 3D^pol^ protein had little effect on virus proliferation in cells (Fig. 1D and E; no significant differences relative to WT). It thus implied that the observed attenuation by these 3A and 3D^pol^ variants in our study may not, at least not mainly, be attributed to the differences in viral replicative speed between them and the WT virus.

### Underlying mechanism of 3A and 3D^pol^ proteins regulating fidelity

Over the past decades, a considerable amount of work has uncovered the molecular determinants of replication fidelity in RdRPs. Unexpectedly, the variation/mutation sites are widely distributed in the RdRP core. It is thus believed that there may exist a more complicated mechanism (41). Here, both V63A and M393L polymerase mutations in EV71 fall into the near sites known to regulate RNA synthesis. At the C-terminus of V63, the residue G64 plays a critical role in the hydrogen bonding network involving the N-terminus and polymerase motif A; its mutation G64S has been documented to be a known high-fidelity mutation of picornaviruses and increases resistance to ribavirin. Additionally, residue M393 is adjacent to the polymerase motif E and forms hydrophobic interactions with motif E residues K376 and R377, which in turn interact with the −1 to −3 backbone region of the product RNA (29). *In vitro* polymerase assay data showed that the V63A + M393L mutation increased nucleotide discrimination, reducing the rate of RNA elongation in both processive elongation and single-nucleotide assays when non-cognate NTP (2’-dATP/NITD008-TP) was provided. Recently, an analogous site of M393V in the polymerase of PV was also reported to confer the high-fidelity phenotype (42), further confirming the important role of this site in fidelity regulation.

In contrast, there is currently no report regarding the 3A/3AB protein in fidelity regulation. Our *in vitro* polymerase assay data demonstrated that 3AB protein, regardless of being WT or V75A mutant, resulted in distinct effects on the fidelity during RNA synthesis in the context of WT and V63A + M393L 3D^pol^ proteins (increase vs. no effect), despite both polymerases exhibiting consistent stimulation by 3AB (Fig. 5). Many studies on the stimulation of 3D^pol^-catalyzed RNA synthesis by 3AB have been conducted on PV. It is thought that 3AB stimulates RNA synthesis through promoting the utilization of 3′-hydroxyl termini as sites for chain elongation by 3D^pol^ (39). During this process, it may require 3AB to interact with and stabilize these sites and/or may recruit 3D^pol^ to the site (39, 43). It has been indicated that many of the binding sites with either RNA or 3D^pol^ are within 3B sequences, although there may exist a synergistic effect between 3A and 3B regions on the 3AB functions (44, 45), and the corresponding contact surface on 3D^pol^ lies in a hydrophobic patch near conserved motif E (24, 45). Thus, it is plausible that the V75A mutation in the 3A sequence had little effect on the stimulation activity of 3AB. In terms of how 3AB, irrespective of the mutation, regulates the fidelity of WT 3D^pol^-catalyzed RNA synthesis, it remains unknown. On the other hand, more work is also needed to determine whether the inability of 3AB to influence the fidelity of RNA synthesis by V63A + M393L 3D^pol^ is attributed to the impairment of 3AB-3D^pol^ interaction caused by the M393L mutation, which resides at the contact surface on 3D^pol^.

It has been reported that 3AB can function as a nucleic acid chaperone with helix-destabilizing activity to facilitate RNA proper folding, although the mechanism is still unclear (28). An *in vitro* helix-unwinding assay was then performed to investigate the effect of the V75A mutation on the RNA chaperone activity of 3AB. Additionally, due to the absence of 3D^pol^ protein, this assay can, in a way, dissect the role of 3A/3AB in fidelity regulation even further, providing a straightforward explanation for the high-fidelity phenotype of the 3A_V75A_ variant. The results showed that despite the V75A mutation being located in the membrane binding region of 3A protein, beyond the reported chaperone functional region (the last 7 C-terminal amino acids of 3A plus the full 3B protein [28]), it was able to cause a reduction in helix-unwinding rate, especially at a low concentration (Fig. 6). In comparison with alanine, valine is a larger hydrophobic residue and thereby has more hydrophobic interaction with membranes. It is often found in the tightly packed hydrophobic core of membrane proteins (46, 47). Analysis of EV71 3A/3AB topology (48, 49) revealed that it is the case for 3A/3AB protein, as there are quite a few Val residues residing in the predicted membrane binding region, implying the essential function of Val residues (Fig. S3). Although it is still unclear regarding the interplay between membrane binding region and chaperone functional region, it seems to be a common event in many RNA viruses that RNA remodeling proteins, like RNA chaperone or helicase, play an important role in fidelity regulation, as demonstrated that the high-fidelity G641D nsP2 of CHIKV (a viral helicase-protease protein) also exhibited a delayed helicase activity (18). Based on such common features shared by EV71 3AB and CHIKV nsP2, the reduction in helix-unwinding activity of V75A 3AB protein may contribute to the enhanced fidelity of 3A_V75A_/3A_V75A_-3D_V63A+M393L_ variants. The detailed mechanism behind the slowed helix-unwinding activity of 3AB-induced high-fidelity phenotype remains to be explored in future work.

Together, our findings have identified that 3A regulates the replication fidelity of EV71. This supports a model in which EV71 uses multiple nonstructural proteins to replicate RNA genomes faithfully. Besides, our data suggest that the 3AB protein with RNA helix-unwinding activity and polymerase may have dynamic interactions to coordinate the replication fidelity of EV71.

## MATERIALS AND METHODS

### Viruses, cells, and compound

Vero cells were cultured in Dulbecco modified Eagle medium (DMEM; Invitrogen) with 10% fetal bovine serum (FBS), 100 U/mL of penicillin, and 100 µg/mL of streptomycin. The WT viruses and variants (designated as 3A_V75A_, 3D_V63A+M393L_, 3A_V75A_-3D_V63A+M393L_, and 3D_F232L_, respectively) were prepared from the infectious cDNA clone of EV71 (29, 50), stored as aliquots at 80°C. The supernatants of transfected cells were harvested at 96 h post-transfection, designated as passage 0 (P0) of the rescued viruses. The P0 was passaged to the second generation (P1), followed by viral titration on Vero cells, and the viruses were stored at 80°C for later use. All viruses (P1) were subject to nucleotide sequencing. NITD008 was synthesized as previously reported (51).

### Deep-sequencing sample preparation and analysis

For multi-step growth curves, Vero cells were infected at MOI = 0.1 and, at different time points, quantified progeny virus by plaque assay. After the P0 generation infected the cells, the viral supernatant of the P1 generation was harvested at 72 h post-infection. The viral RNA was extracted using the QIAamp Viral RNA Mini Kit (Qiagen, Hilden, Germany) and quantified by quantitative reverse transcription-PCR (qRT-PCR) using VP1 primers (Table 2). Then, the RNA was converted to double-stranded DNA prior to generating a compatible sequencing library using the Nextera XT DNA Library Preparation kit. NGS was performed on the DNBSEQ T7 sequencing platform (MGI, Shenzhen, China).

**TABLE 2:** Sequences of all primers

Primer set	Forward primer	Forward primer sequence (5’ to 3’)^*a*^	Reverse primer	Reverse primer sequence (5’ to 3’)
1	VP1-F	AGATAGGGTGGCAGATGTAATTGAAAG	VP1-R	TAGCATTTGATGATGCTCCAATTTCAG
2	15F	GGGTTGCACCCACTCACAGG	2009R	GGTGGATTGCCATGGTCCAT
3	41F	AATAATGTGCCCACGAATGC	3846R	TTGAGACTGCGTCAGTGAAG
4	3601F	CAGCCAGGTACCAATCACAT	5600R	TTCCAGGTTGACTCCTTGCT
5	5392F	CGAGCCTTGACTTTGCTCTC	7633R	AAATTTACCCCCACCAGTCACATT
6	RNA1	*CAUUAUCGGAUAGUGGAA CCUAGCUUCGACUAUGGAU AAUC		
7	RNA2	AAUAAAGAUUAUCCGAUAGU CGAAGCUAGGUUCCACUAUC CGAUAAUGAAAUAA		
8	3A-F	AAACGTTGCAGCCCATTAGT	3A-R	AATTTTATCAATCGAGCGCAG
9	3D-F	CACTTATGTCAAGGACGAGCTG	3D-R	CTAGAAGCTTTTTTTTTTTTTTTTTTTTTT

^*a*^ *, HEX labeled site.

Sequencing runs were analyzed using the ViVan bioinformatics pipeline. SNP detection was performed with default parameters, except that the minimum coverage was set to 3,000, minimum variant frequency was set to 1.0%, and the ploidy was set to 1 (13, 52, 53). For each position throughout the viral genome, base composition and frequency were counted to calculate the number of variants for the whole genome, each protein, and (non-)synonymous mutation.

To calculate RMSD for each nucleotide position, the RMSD formula (54) was modified to compare the distribution of nucleotides between each sample by PHDtools (55), which were previously utilized for analyses of population diversity (56–58): RMSD (X, Y) i = $\sqrt{\frac{\sum p=\{A,T,G,C,-,I\}{\left(P\left[X_{i,p}\right]-P\left[Y_{i,p}\right]\right)}^{2}}{6}}$. P[Xi,p] and P[Yi,p] are the probabilities of nucleotide p, at position i, for the samples X and Y, respectively. The nucleotide, p, is an element of the nucleotide set [A, T, G, C, -, I], where the - and I characters represent deletion and insertion. The denominator within the square root operator, 6, is the number of symbols in the nucleotide set used.

### Construction of recombinant baculoviruses

The cDNAs for EV71 3AB from the plasmid containing full-length EV71 cDNA (29, 50), and MBP fragments were amplified by polymerase chain reaction (PCR), followed by cloning into the vector pFastBac. The desired plasmids were subjected to the Bac-to-Bac baculovirus system to express the recombinant proteins with an MBP fused at the N-terminus as previously described (28).

### Protein expression and purification

The pET26b-Ub vector-based plasmid containing the EV71 3D^pol^ (RdRP) gene (50) was used as the original cloning template to construct the mutant plasmids according to previously described methods, designated as the EV71 3D^pol^ WT and V63A + M393L 3D^pol^, respectively (59). Cell growth, isopropyl-β-D-thiogalactopyranoside (IPTG) induction, cell harvesting, cell lysis, protein purification, and protein storage were performed as previously described (60).

The expression and purification of MBP alone and MBP-3AB proteins were performed as previously described (61). Briefly, Spodoptera frugiperda insect cells (Sf9) were infected with the recombinant baculoviruses and harvested at 72 h postinfection. Cell pellets were resuspended, lysed by sonication, and subjected to centrifugation for 30 min at 11,000 × *g* to remove debris. The protein in the supernatant was purified using amylase affinity chromatography (New England BioLabs, Ipswich, MA) according to the protocol and then further purified by Superdex 75 column in the AKTA system.

### Primer-dependent polymerase assay

For the primer-dependent polymerase assay of EV71 3D^pol^, a 50 μL-reaction mixture containing 6 µM 3D^pol^ or its variant, 4 µM RNA construct (T33/P10) (34, 62), a certain NTP/NITD008-TP substrate combination in buffer (50 mM Tris-HCl [pH 7.0], 75 mM KCl, 5 mM MgCl_2_, 4 mM tris (2-carboxyethyl) phosphine [TCEP]) was incubated at 22.5°C. In some reactions, viral protein 3AB was added at the indicated concentration during the pre-incubation. At different times, an 8 µL portion of the reaction mixture was withdrawn and quenched with an equal volume of stop solution (90% [vol/vol] formamide, 10 mM EDTA (pH 8), bromophenol blue, and xylene cyanol, 0.1% [wt/vol] each), and the products were analyzed on a 20% polyacrylamide (wt/vol)/7 M urea gel electrophoresis. RNA species were visualized by Stains-All (Sigma-Aldrich) staining and quantified by ImageJ software. The relative intensity was plotted as a function of time and fit to a single exponential equation by GraphPad Prism 7.0 (GraphPad Software, Inc., San Diego, CA), Y = C + amplitude [1 - exp (-*k*_obs_ × t)], where Y is the relative intensity, *k*_obs_ is the observed rate constant for nucleotide incorporation, t is the time and C is an offset.

### Elongation complex (EC) purification and the EC extension assay

EC assembly and purification were performed as previously described (36). Briefly, the EC was assembled using the T33/P10 and EV71 RdRP upon incorporation of a G nucleotide with GTP as the only NTP substrate, producing an 11-mer (P11)-containing EC (EC11) and purified using a Capto HiRes Q column (GE Healthcare). The final buffer condition for EC storage was 20 mM HEPES [pH 7.0], 100 mM NaCl, 2 mM MgCl_2_, and 4 mM TCEP.

The EC elongation experiments were performed in a 50 µL reaction mixture containing 4 µM purified EC, 300 µM NITD008-TP at 10°C. Aliquots (8 µL) were withdrawn and quenched with an equal volume of stop solution at the different time points. RNA bands were visualized using Stains-All (Sigma-Aldrich) staining and quantified by ImageJ software.

### Nucleic acid helix unwinding assay

The standard helix destabilizing assay was performed as previously described (61). RNA helix substrates were prepared by annealing two complementary nucleic acid strands, RNA1 and RNA2 (Table 2). Briefly, different concentrations of proteins and 0.1 pmol of helix substrate were added to a mixture containing a final concentration of 25 mM HEPES-KOH (pH 8.0), 50 mM NaCl, 1 mM MgCl_2_, 5 U RNasin (Promega), and 10 mM ATP and incubated at 37°C for different times. Mixtures were electrophoresed on 12% native-PAGE gels. Gels were scanned with a Typhoon 9200 imager (GE Healthcare). The ratio of released single strands versus the total substrates was quantified with ImageJ software.

### Animal experiment

The progenies of the pregnant ICR mice were assigned randomly to five groups, and each group had 10 newborn mice. Two-day-old ICR newborn mice (*n* = 10) were inoculated intraperitoneally with viruses/virus-free cell culture supernatant at a concentration of 10^7^ PFU/ mouse. The suckling mice were monitored daily for body weight, clinical symptoms, and mortality for 20 days. To minimize animal suffering, the mice were euthanized if they were quadriplegic. Mice from each treatment group were euthanized on 4 dpi, and the brain, muscle, liver, lung, spleen, and small intestine were collected. Samples were homogenized by using a TissueLyser LT homogenizer (Qiagen) in DMEM. The virus titers in the supernatants of clarified homogenates (3,000 × *g* for 10 min at 4°C) were determined by plaque assay. The statistical analyses of virus titers were performed using one-way ANOVA, and *P* values of 0.05 were considered significant.

For NGS samples from brain and muscle tissues, target enrichment on equivalent quantities of viral RNA was performed by high-fidelity RT-PCR (Accuscript PfuUltra II) of four cDNA amplicons spanning the EV71 5′ to 3′ UTRs (Table 2). The follow-up procedure was the same as above.

### Direct competition fitness assay

For direct competition fitness assays, each variant was mixed with the WT virus at a ratio of 1:1 to infect Vero cells in triplicate wells at an MOI of 0.1 over three passages. Viral RNA was extracted, and the region flanking 3A amino acid 75 and 3D amino acid 63 or 393 was amplified by RT-PCR for Sanger sequencing. The abundance of each competitor was measured as the height of the nucleotide encoding either the WT (3A_V75A_-GTG; 3D_V63A_-GTG; 3D_M393L_-ATG) or variants (3A_V75A_-GCG; 3D_V63A_-GCG; 3D_M393L_-TTG) in sequencing chromatograms and quantified by ImageJ software. The primers used in the direct competition assays are listed in Table 2.

## Data Availability

The raw sequence data reported in this paper have been deposited in the Genome Sequence Archive (63) in National Genomics Data Center (64), China National Center for Bioinformation / Beijing Institute of Genomics, Chinese Academy of Sciences (GSA: CRA016439).

## SUPPLEMENTAL MATERIAL

Supplemental material jvi.01207-25-s0001.pdf Figures S1 to S3; Table S1.

ASM does not own the copyrights to Supplemental Material that may be linked to, or accessed through, an article. The authors have granted ASM a non-exclusive, world-wide license to publish the Supplemental Material files. Please contact the corresponding author directly for reuse.
