# Supplementary material for: Mutation in enterovirus 71 nonstructural protein 3A increases genome replication fidelity and exhibits attenuated virulence in mice
Source: J Virol. 2025 Sep 17;99(10):e01207-25. doi: 10.1128/jvi.01207-25 (PMC12548465; doi:10.1128/jvi.01207-25)
Supplement: Supplemental material — Figures S1 to S3; Table S1. [file jvi.01207-25-s0001.pdf]

S1

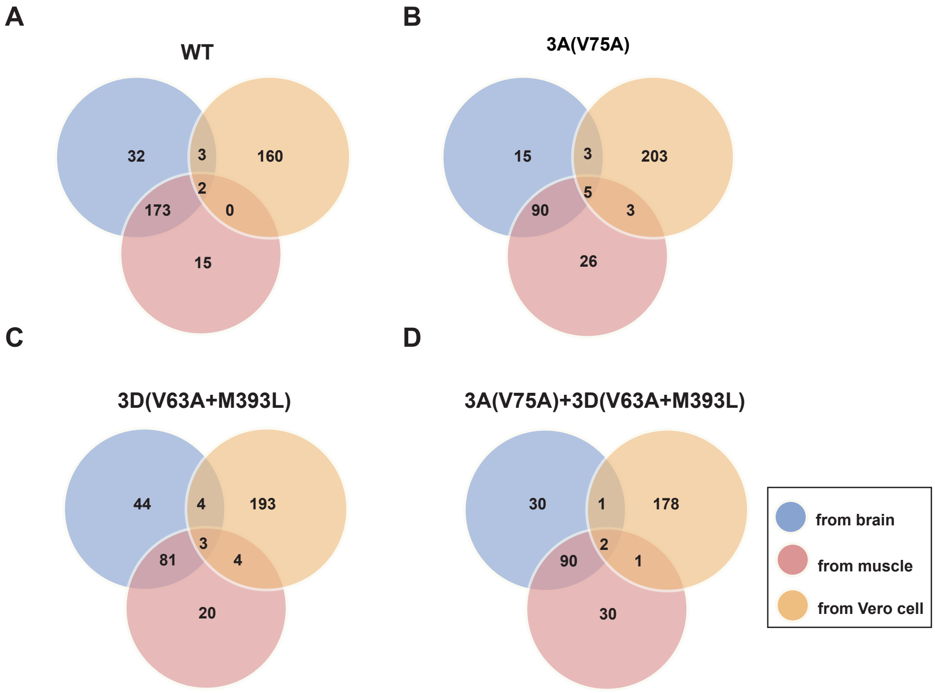

A
WT
32
3
160
2
173
0
15
B
3A(V75A)
15
3
203
5
90
3
26
C
3D(V63A+M393L)
44
4
193
3
81
4
20
D
3A(V75A)+3D(V63A+M393L)
30
1
178
2
90
1
30
from brain
from muscle
from Vero cell

FIG S1 The comparison of 0.5-1% SNPs isolated from mice and Vero cells. (A-D) The number of 0.5-1% SNPs produced by WT, $3A_{V75A}$, $3D_{V63A+M393L}$ and $3A_{V75A}$-$3D_{V63A+M393L}$ EV71 in mouse brain, mouse muscle and Vero cells.

S2

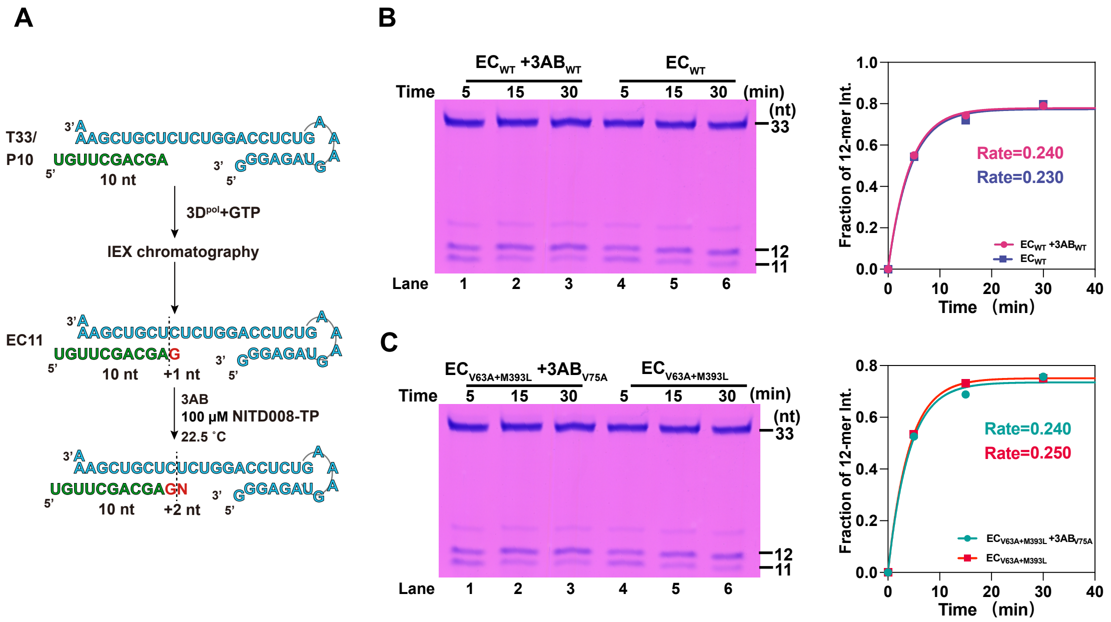
A
T33/
P10
AAAGCUGCUCUCUGGACCUCUG
UGUUCGACGA
GGGAGAUG
10 nt
3Dpol+GTP
IEX chromatography
EC11
UGUUCGACGAG
10 nt
+1 nt
3AB
100 μM NITD008-TP
22.5 °C
UGUUCGACGAGN
+2 nt
B
ECWT +3ABWT
ECWT
Time
5 15 30 5 15 30 (min)
(nt)
33
12
11
Lane
1 2 3 4 5 6
Fraction of 12-mer Int.
Rate=0.240
Rate=0.230
ECWT +3ABWT
ECWT
Time (min)
C
ECV63A+M393L +3ABV75A
ECV63A+M393L
Rate=0.240
Rate=0.250
ECV63A+M393L +3ABV75A
ECV63A+M393L

FIG S2 The effect of 3AB for single-nucleotide incorporation assay of EC. (A) The reaction flow chart and products of polymerase assays. (B/C) The elongation products accumulation is monitored over time for the WT EC and V63A+M393L EC with or without 3AB. The relative 12 nt-product intensity (calculated as the intensity of products of 12 divided by the total intensity of products and primers) as a function of time is plotted for all constructs under GTP substrate.

S3

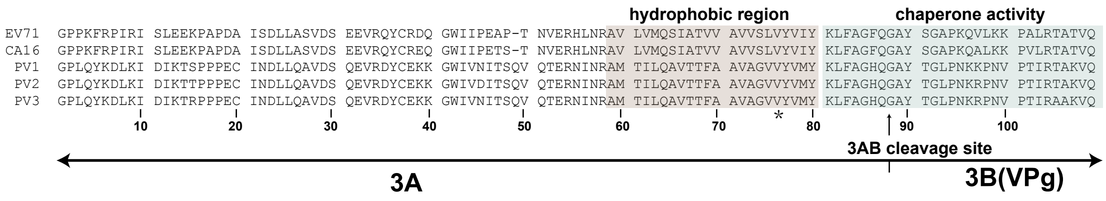
hydrophobic region
chaperone activity
EV71 GPPKFRPIRI SLEEKPAPDA ISDLLASVDS EEVRQYCRDQ GWIIPEAP-T NVERHLNRAV LVMQSIATVV AVVSLVYVIY KLFAGFQGAY SGAPKQVLKK PALRTATVQ
CA16 GPPKFRPIRI SLEEKPAPDA ISDLLASVDS EEVRQYCREQ GWIIPETS-T NVERHLNRAV LVMQSIATVV AVVSLVYVIY KLFAGFQGAY SGAPKQALKK PVLRTATVQ
PV1 GPLQYKDLKI DIKTSPPPEC INDLLQAVDS QEVRDYCEKK GWIVNITSQV QTERNINRAM TILQAVTTFA AVAGVVYVMY KLFAGHQGAY TGLPNKKPNV PTIRTAKVQ
PV2 GPLQYKDLKI DIKTTPPPEC INDLLQAVDS QEVRDYCEKK GWIVDITSQV QTERNINRAM TILQAVTTFA AVAGVVYVMY KLFAGHQGAY TGLPNKRPNV PTIRTAKVQ
PV3 GPLQYKDLKI DIKTRPPPEC INDLLQAVDS QEVRDYCEKK GWIVNITSQV QTERNINRAM TILQAVTTFA AVAGVVYVMY KLFAGHQGAY TGLPNKRPNV PTIRAAKVQ
10
20
30
40
50
60
70
*
80
90
100
3AB cleavage site
3A
3B(VPg)

FIG S3 Amino acid sequence alignments of the 3AB regions of various members of the enterovirus. The boxed region indicates the well conserved 22-amino acid hydrophobic domain (aa 59–80) and chaperone activity (aa 81–109). Asterisks (*) indicate the mutation site of 3A protein in this study, while dash (-) denote a gap used solely to maintain the alignment. GenBank numbers of the enterovirus are JX678881, KC117317, V01149, M12197 and K01392.

Table S1. SNPs at >1% frequency of WT, $3A_{V75A}$, $3D_{V63A+M393L}$, and $3A_{V75A}$-$3D_{V63A+M393L}$ EV71 from Vero cells.

WT				$3A_{V75A}$				$3D_{V63A+M393L}$				$3A_{V75A}$-$3D_{V63A+M393L}$			
Pos	Gene	Depth	nt sub	Pos	Gene	Depth	nt sub	Pos	Gene	Depth	nt sub	Pos	Gene	Depth	nt sub
335	5'UTR	310261	T-A	345	5'UTR	353997	G-T	345	5'UTR	142503	G-T	345	5'UTR	190476	G-T
336	5'UTR	312830	T-C	348	5'UTR	351417	A-C	348	5'UTR	141607	A-C	348	5'UTR	189014	A-C
338	5'UTR	317998	C-G	372	5'UTR	339211	G-A	355	5'UTR	141051	C-G	372	5'UTR	181800	G-A
345	5'UTR	318684	G-T	373	5'UTR	339506	C-G	372	5'UTR	136504	G-A	373	5'UTR	181892	C-G
348	5'UTR	319089	A-C	375	5'UTR	340809	T-G	373	5'UTR	136501	C-G	375	5'UTR	182410	T-G
355	5'UTR	321214	C-G	376	5'UTR	344910	G-C	375	5'UTR	136630	T-G	376	5'UTR	184735	G-C
372	5'UTR	330827	G-A	385	5'UTR	353906	G-T	376	5'UTR	138363	G-C	385	5'UTR	189798	G-T
373	5'UTR	334050	C-G	388	5'UTR	355033	A-C	385	5'UTR	141259	G-T	388	5'UTR	190227	A-C
375	5'UTR	336601	T-G	394	5'UTR	363255	G-GAP	388	5'UTR	141700	A-C	394	5'UTR	194641	G-GAP
376	5'UTR	342407	G-C	434	5'UTR	414097	G-A	394	5'UTR	144661	G-GAP	434	5'UTR	222880	G-A
385	5'UTR	352958	G-T	438	5'UTR	427712	G-I	434	5'UTR	167630	G-A	440	5'UTR	231168	T-C
388	5'UTR	354911	A-C	440	5'UTR	427875	T-C	438	5'UTR	173624	G-I	535	5'UTR	395501	C-A
394	5'UTR	364122	G-GAP	446	5'UTR	441983	T-A	440	5'UTR	174145	T-C	538	5'UTR	410672	A-I
395	5'UTR	366547	G-C	496	5'UTR	606924	A-T	446	5'UTR	180551	T-A	541	5'UTR	411187	C-G
396	5'UTR	366044	G-A	503	5'UTR	626207	C-T	535	5'UTR	290013	C-A	545	5'UTR	417417	T-A
397	5'UTR	363970	G-T	535	5'UTR	706505	C-A	538	5'UTR	299746	A-I	546	5'UTR	420910	A-C
434	5'UTR	369900	G-A	538	5'UTR	729530	A-I	545	5'UTR	305439	T-A	548	5'UTR	433382	T-C
438	5'UTR	365219	G-I	545	5'UTR	742356	T-A	546	5'UTR	307816	A-C	549	5'UTR	434605	T-A
440	5'UTR	362983	T-C	546	5'UTR	748241	A-C	548	5'UTR	316647	T-C	550	5'UTR	433538	T-A
446	5'UTR	369876	T-A	548	5'UTR	770458	T-C	549	5'UTR	317465	T-A	551	5'UTR	433717	G-A
496	5'UTR	518221	A-T	549	5'UTR	772477	T-A	550	5'UTR	316918	T-A	553	5'UTR	439748	G-T
500	5'UTR	528702	C-T	550	5'UTR	770433	T-A	551	5'UTR	316999	G-A	554	5'UTR	443268	T-A
503	5'UTR	529304	C-T	551	5'UTR	770795	G-A	553	5'UTR	321874	G-T	558	5'UTR	458407	C-G
535	5'UTR	539008	C-A	553	5'UTR	781277	G-T	554	5'UTR	323904	T-A	595	5'UTR	540115	G-A
538	5'UTR	539473	A-I	554	5'UTR	786160	T-A	558	5'UTR	334326	C-G	602	5'UTR	561270	A-C
545	5'UTR	539998	T-A	558	5'UTR	811041	C-G	595	5'UTR	388238	G-A	603	5'UTR	570044	A-T
546	5'UTR	537651	A-C	595	5'UTR	954222	G-A	602	5'UTR	401264	A-C	604	5'UTR	570202	A-T
548	5'UTR	539876	T-C	602	5'UTR	990048	A-C	603	5'UTR	407675	A-T	605	5'UTR	570774	G-T
549	5'UTR	531298	T-A	603	5'UTR	1005043	A-T	604	5'UTR	407742	A-T	612	5'UTR	576580	T-C
550	5'UTR	538732	T-A	604	5'UTR	1005394	A-T	605	5'UTR	407930	G-T	1402	VP2	256316	C-T
551	5'UTR	531273	G-A	605	5'UTR	1005756	G-T	612	5'UTR	412000	T-C	1403	VP2	245235	T-G
553	5'UTR	530082	G-T	612	5'UTR	1016144	T-C	967	VP2	353116	C-G	1408	VP2	242796	C-T
554	5'UTR	538909	T-A	963	VP2	914714	G-C	971	VP2	352644	T-G	1411	VP2	244056	G-A
558	5'UTR	549321	C-G	967	VP2	899708	C-G	976	VP2	350754	A-T	1412	VP2	243276	C-A
595	5'UTR	718300	G-A	971	VP2	898090	T-G	1402	VP2	199164	C-T	1416	VP2	246968	G-A
602	5'UTR	737416	A-C	976	VP2	893700	A-T	1403	VP2	191553	T-G	1417	VP2	247950	A-T

603	5'UTR	749773	A-T	1402	VP2	541786	C-T	1408	VP2	190048	C-T	1418	VP2	248108	T-C
604	5'UTR	750029	A-T	1403	VP2	518233	T-G	1411	VP2	191471	G-A	1422	VP2	257450	T-G
605	5'UTR	751702	G-T	1408	VP2	513215	C-T	1412	VP2	190839	C-A	1423	VP2	257218	T-C
612	5'UTR	759930	T-C	1411	VP2	515740	G-A	1416	VP2	192752	G-A	1426	VP2	262012	A-G
948	VP4	563270	A-T	1412	VP2	514689	C-A	1417	VP2	192947	A-T	1666	VP2	307325	C-G
950	VP4	565462	G-GAP	1416	VP2	520251	G-A	1418	VP2	193147	T-C	1667	VP2	306422	C-G
954	VP2	570491	C-A	1417	VP2	520872	A-T	1422	VP2	198601	T-G	2136	VP3	259445	C-T
958	VP2	570889	C-G	1418	VP2	520972	T-C	1423	VP2	198458	T-C	2137	VP3	260435	T-G
960	VP2	578021	G-GAP	1422	VP2	537582	T-G	1426	VP2	201830	A-G	2139	VP3	262779	C-G
963	VP2	577907	G-C	1423	VP2	536950	T-C	1666	VP2	236941	C-G	2141	VP3	264089	C-A
965	VP2	585040	G-I	1426	VP2	547193	A-G	1667	VP2	236380	C-G	2142	VP3	262945	A-G
967	VP2	569234	C-G	1666	VP2	618921	C-G	2052	VP3	199195	T-C	2149	VP3	264167	G-T
971	VP2	567081	T-G	1667	VP2	617378	C-G	2129	VP3	195513	A-C	2154	VP3	264347	A-G
974	VP2	577943	A-I	2048	VP3	530593	A-C	2136	VP3	196574	C-T	2155	VP3	267734	C-T
976	VP2	562712	A-T	2129	VP3	512525	A-C	2137	VP3	197174	T-G	2480	VP1	309297	T-G
1402	VP2	458825	C-T	2136	VP3	516583	C-T	2139	VP3	198756	C-G	2489	VP1	311995	C-GAP
1403	VP2	448321	T-G	2137	VP3	518477	T-G	2141	VP3	199801	C-A	2490	VP1	310697	A-T
1408	VP2	444648	C-T	2139	VP3	523038	C-G	2142	VP3	198912	A-G	2494	VP1	311761	C-G
1411	VP2	448716	G-A	2141	VP3	526133	C-A	2149	VP3	199773	G-T	2495	VP1	310421	C-G
1412	VP2	448404	C-A	2142	VP3	523998	A-G	2154	VP3	200227	A-G	2498	VP1	317420	C-I
1416	VP2	449322	G-A	2149	VP3	526009	G-T	2155	VP3	202648	C-T	2499	VP1	309244	A-G
1417	VP2	448378	A-T	2154	VP3	527713	A-G	2480	VP1	239771	T-G	2590	VP1	327022	A-G
1418	VP2	449241	T-C	2155	VP3	534172	C-T	2489	VP1	242592	C-GAP	2955	VP1	200506	A-C
1422	VP2	454739	T-G	2480	VP1	670009	T-G	2490	VP1	241662	A-T	3219	VP1	167733	T-A
1423	VP2	454438	T-C	2489	VP1	679302	C-GAP	2494	VP1	242883	C-G	3222	VP1	164543	A-T
1426	VP2	460532	A-G	2490	VP1	676940	A-T	2495	VP1	241792	C-G	3223	VP1	164060	T-A
2126	VP3	397528	A-C	2494	VP1	679910	C-G	2498	VP1	248282	C-I	3224	VP1	163262	A-T
2127	VP3	401233	G-C	2495	VP1	676200	C-G	2499	VP1	241409	A-G	3227	VP1	164549	T-A
2128	VP3	404029	G-C	2498	VP1	692764	C-I	2590	VP1	259518	A-G	3232	VP1	166612	C-I
2129	VP3	406973	A-C	2499	VP1	673453	A-G	3219	VP1	132860	T-A	3713	2A	160625	C-G
2136	VP3	405573	C-T	2590	VP1	724426	A-G	3222	VP1	130117	A-T	4232	2C	261913	G-C
2137	VP3	406447	T-G	2955	VP1	423363	A-C	3223	VP1	129783	T-A	4699	2C	302049	A-T
2139	VP3	410025	C-G	3219	VP1	358117	T-A	3224	VP1	129196	A-T	4705	2C	305428	G-A
2141	VP3	411434	C-A	3222	VP1	351092	A-T	3227	VP1	130427	T-A	4707	2C	306803	G-A
2142	VP3	409964	A-G	3223	VP1	350143	T-A	3232	VP1	132059	C-I	4708	2C	303843	T-C
2153	VP3	414887	G-T	3224	VP1	348248	A-T	3713	2A	120962	C-G	4710	2C	304067	T-C
2154	VP3	416872	A-G	3227	VP1	351847	T-A	4232	2C	198557	G-C	4716	2C	297866	A-T
2335	VP3	568213	C-I	3232	VP1	356841	C-I	4699	2C	226381	A-T	6860	3D	34455	A-G
2344	VP3	552700	C-T	3713	2A	342047	C-G	4705	2C	228989	G-A				

2345	VP3	555357	A-G	4232	2C	525481	G-C	4707	2C	230236	G-A
2480	VP1	482002	T-G	4699	2C	645538	A-T	4708	2C	228178	T-C
2489	VP1	488185	C-GAP	4705	2C	653244	G-A	4710	2C	228681	T-C
2490	VP1	486267	A-T	4707	2C	657359	G-A	4716	2C	223929	A-T
2494	VP1	489189	C-G	4708	2C	650652	T-C	6860	3D	26221	A-G
2495	VP1	485975	C-G	4710	2C	651831	T-C				
2498	VP1	495972	C-I	4716	2C	640048	A-T				
2499	VP1	482807	A-G	6860	3D	64209	A-G				
2590	VP1	498763	A-G								
2955	VP1	480921	A-C								
3219	VP1	273468	T-A								
3222	VP1	268771	A-T								
3223	VP1	268004	T-A								
3224	VP1	267343	A-T								
3227	VP1	269230	T-A								
3232	VP1	279731	C-I								
3713	2A	478392	C-G								
4232	2C	472931	G-C								
4398	2C	473920	A-G								
4699	2C	473652	A-T								
4705	2C	479032	G-A								
4707	2C	475332	G-A								
4708	2C	472940	T-C								
4710	2C	473927	T-C								
4716	2C	473339	A-T								
5515	3C	456857	C-A								
5520	3C	461311	A-G								
6860	3D	50800	A-G								

Pos, postion; nt sub, nucleotide substitution.
